# Supplementary material for: Synthesis of Tris-pillar[5]arene and Its Association with Phenothiazine Dye: Colorimetric Recognition of Anions
Source: Molecules. 2019 May 10;24(9):1807. doi: 10.3390/molecules24091807 (PMC6539510; doi:10.3390/molecules24091807)

Synthesis of tris-pillar[5]arene and its association with phenothiazine dye: colorimetric recognition of anions

Alena Khadieva, Vladimir Gorbachuk, Dmitriy Shurpik and Ivan Stoikov *

Kazan Federal University, Kremlevskaya, 18, Kazan, Russian Federation; public.mail@kpfu.ru

Table of Contents:

Synthesis of 3,7-bis(phenylamino)phenothiazin-5-ium iodide

Synthesis of N-phenyl-3-(phenylimino)-3H-phenothiazin-7-amine

Figure 1S. MALDI mass-spectrum (matrix: 2,5-dihydroxybenzoic acid) of 4

Figure 2S. FTIR-ATR spectrum of 4

Figure 3S. ^{1}H NMR (DMSO d_6) spectrum of 4

Figure 4S. ^{13}C NMR (DMSO d_6) spectrum of 4

Figure 5S. ^{1}H NMR ($CDCl_3$) spectra of PhTz (red), 4:PhTz complex (blue) and 4 (black).

Figure 6S. Fragments of the ^{1}H NMR ($CDCl_3$) spectra of PhTz (red), 4:PhTz complex (blue) and 4 (black), sections corresponding to signal of aromatic protons (left), ~OCH2CONH~ fragment connecting pillar[5]arene to TREN (center) and $N(CH_2CH_2NH)$ protons (right).

Figure 7S. Figure 7S. ^{1}H NMR (DMSO d_6) spectra of PhTz (red), 4:PhTz complex (blue) and 4 (black), section corresponding to signal of amide fragment (left).

Figure 8S. ^{31}P NMR ($CDCl_3$) spectra of tetrabutylammonium dyhydrogen phosphate ($C=5\times10^{-3}$) (a), mixture of tetrabutylammonium dyhydrogen phosphate and 4 ($C=5\times10^{-3}$) (b) and a mixture of tetrabutylammonium dyhydrogen phosphate, 4, PhTz ($C=5\times10^{-3}$) (c)

Figure 9S. ^{1}H NMR (DMSO d_6) spectrum of 3,7-bis(phenylamino)phenothiazin-5-ium iodide, sections corresponding to NH (left) and Ar-H (right) protons

Figure 10S. ^{1}H NMR (DMSO d_6) spectrum of N-phenyl-3-(phenylimino)-3H-phenothiazin-7-amine (PhTz)

Synthesis of 3,7-bis(phenylamino)phenothiazin-5-ium iodide

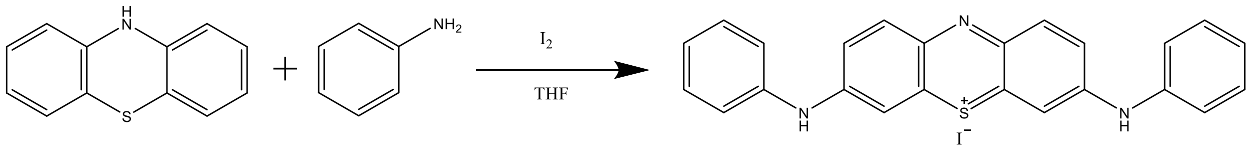

Phenothiazine (1 g, 5.0 mmol) was dissolved in 50 ml of THF at 0°C. Iodine solution in THF was dropwise added to the reaction mixture within 30 min. Then, aniline solution (4.6 g, 50.0 mmol in THF) was dropwise added to the reaction mixture within 15 min. The reaction mixture was stirred for 24 h. The sedimented product was separated by filtration and washed with diethyl ether. The dark blue powder was collected and dried overnight in a vacuum desiccator over P_2O_5 (yield 80%).

^{1}H NMR (400 MHz, DMSO-d_6): δ 11.06 (s, 2H), 8.12 (d, J = 9.3 Hz, 2H), 7.62 – 7.51 (m, 8H), 7.47 (d, J = 7.5 Hz, 4H), 7.37 (t, J = 7.3 Hz, 2H).

Elemental analysis: calculated for $C_{24}H_{18}N_3S^+$: C, 75.76; H, 4.77; N, 11.04; S, 8.43, found: C, 75.70; H, 4.81; N, 11.02; S, 8.42.

Synthesis of N-phenyl-3-(phenylimino)-3H-phenothiazin-7-amine

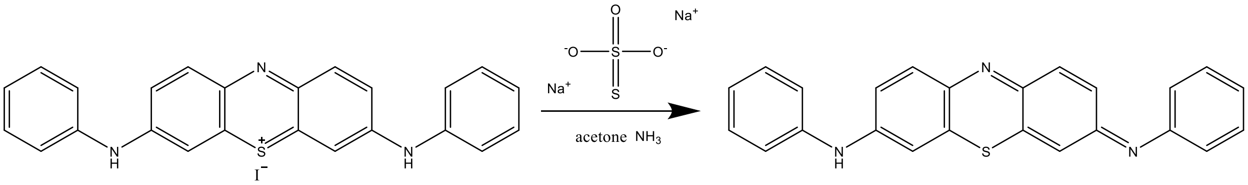

3,7-bis(phenylamino)phenothiazin-5-ium iodide (1 g, 20 mmol) was dissolved in 50 ml of acetone. Then, sodium thiosulfate (6.32 g, 40.0 mmol) and 2% aqueous ammonia were added to the reaction mixture. The reaction mixture was stirred for 12 h. Purple powder was collected and dried overnight in vacuum desiccator over P_4O_{10} (yield 70%).

^{1}H NMR (400 MHz, $(CD_3)_2SO$): δ 9.08 (d, J = 8.6 Hz, 1H), 7.54-7.46 (m, 1H), 7.41-7.33 (m, 4H), 7.26-7.18 (m, 3H), 7.12 (t, J = 7.9 Hz, 1.5H), 7.09 – 7.01 (m, 2.5H), 7.01 – 6.93 (m, 1H), 6.93-6.79 (m, 2.5H), 6.53 (d, J = 1.9 Hz, 0.5H).

Elemental analysis: calculated for $C_{24}H_{17}N_3S$: C, 75.96; H, 4.52; N, 11.07; S, 8.45, found: C, 75.87; H, 4.50; N, 11.04; S, 8.42.

Melting point: 215-217°C (literature 215-216°C) [28].

Figure 1S. MALDI mass-spectrum (matrix: 2,5-dihydroxybenzoic acid) of 4

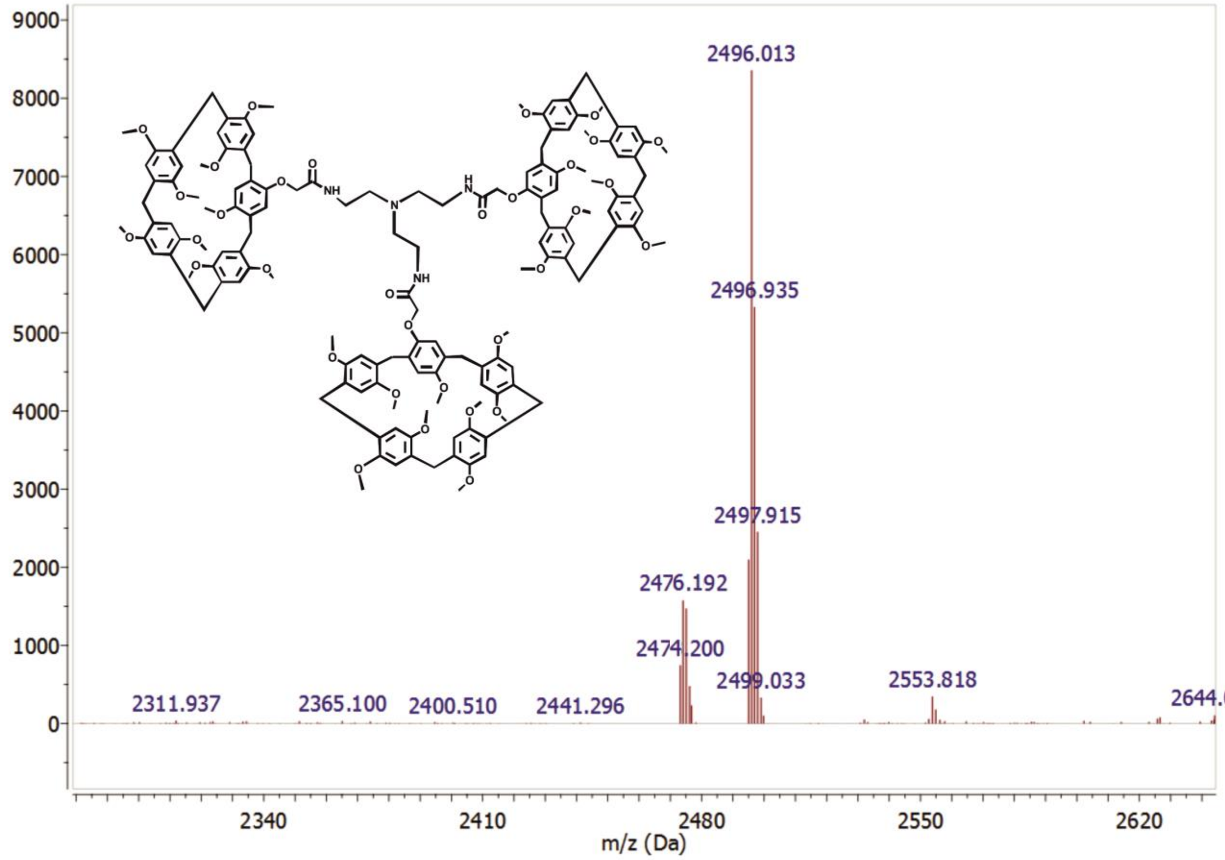

Figure 2S. FTIR-ATR spectrum of 4

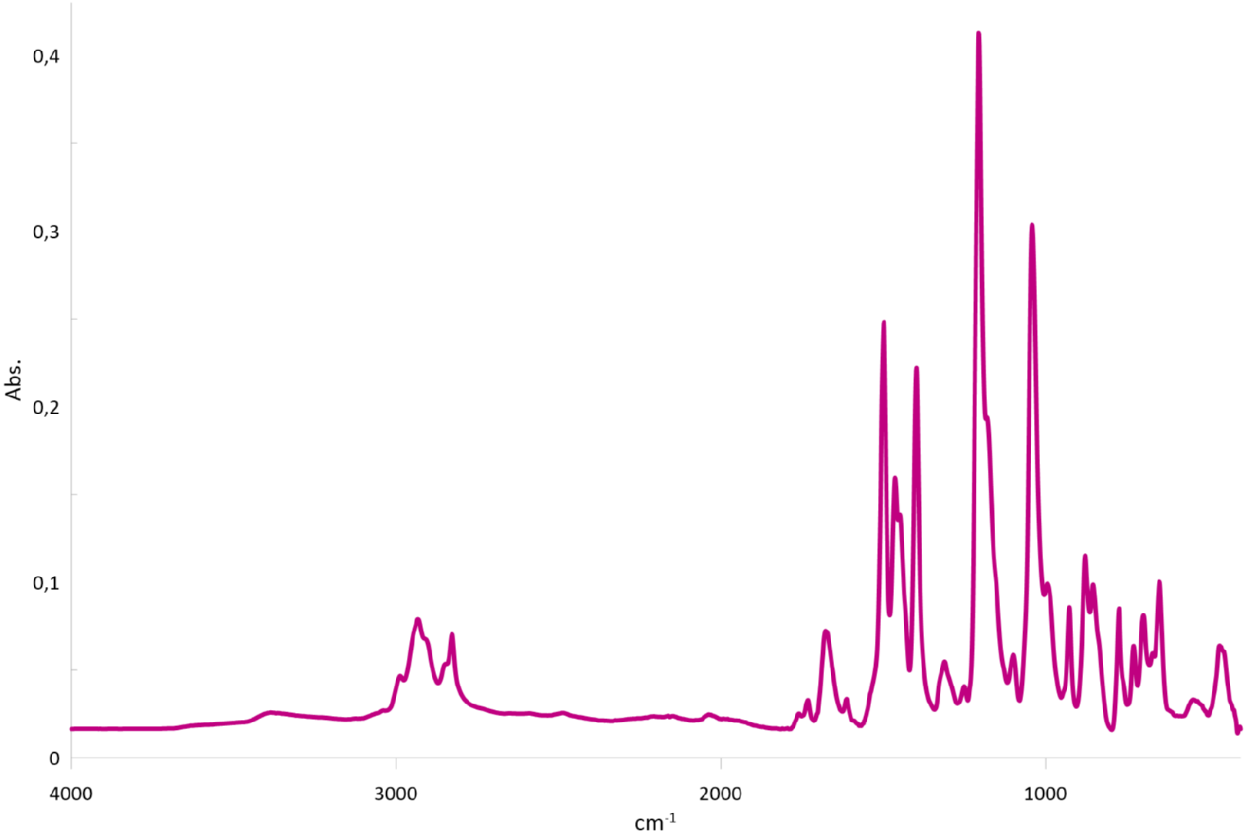

Figure 3S. ^{1}H NMR (DMSO d_6) spectrum of 4

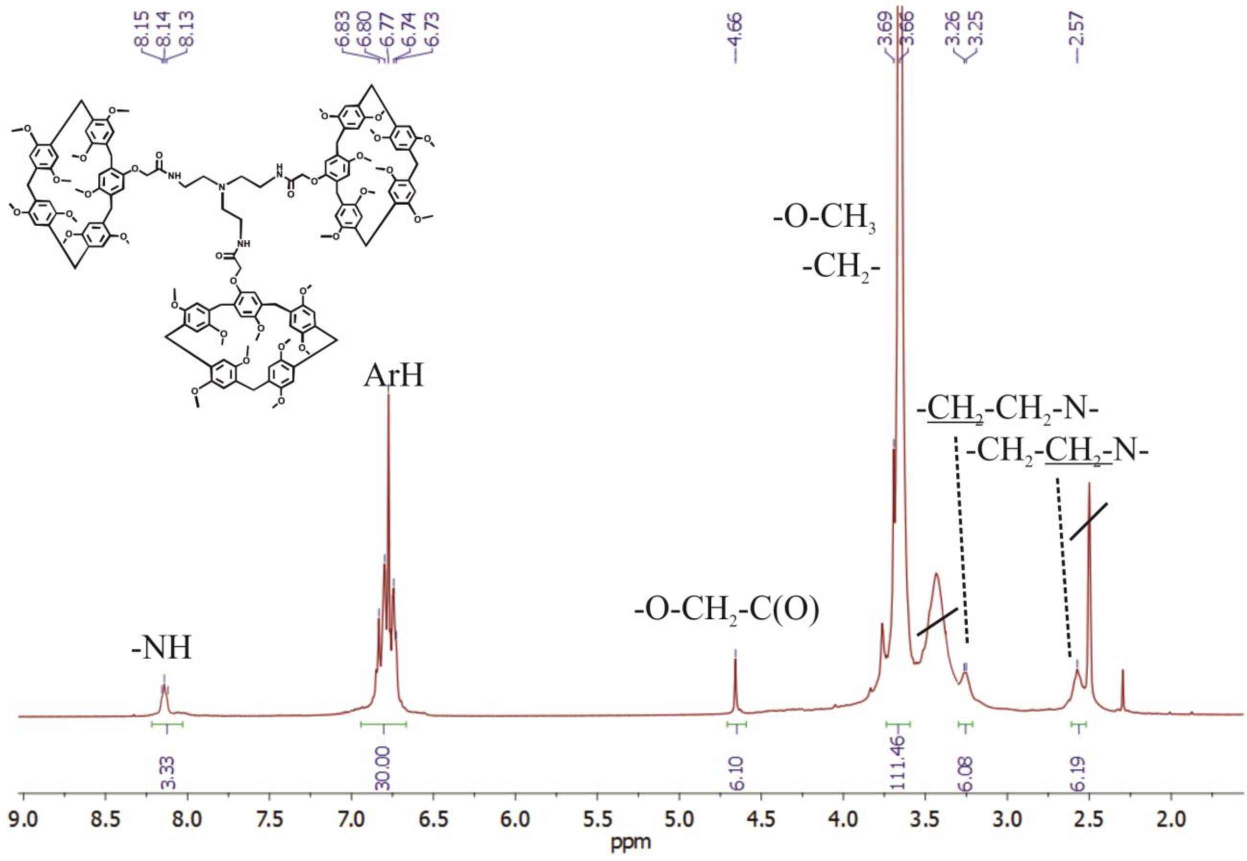

Figure 4S. ^{13}C **NMR (DMSO** d_6**) spectrum of 4**

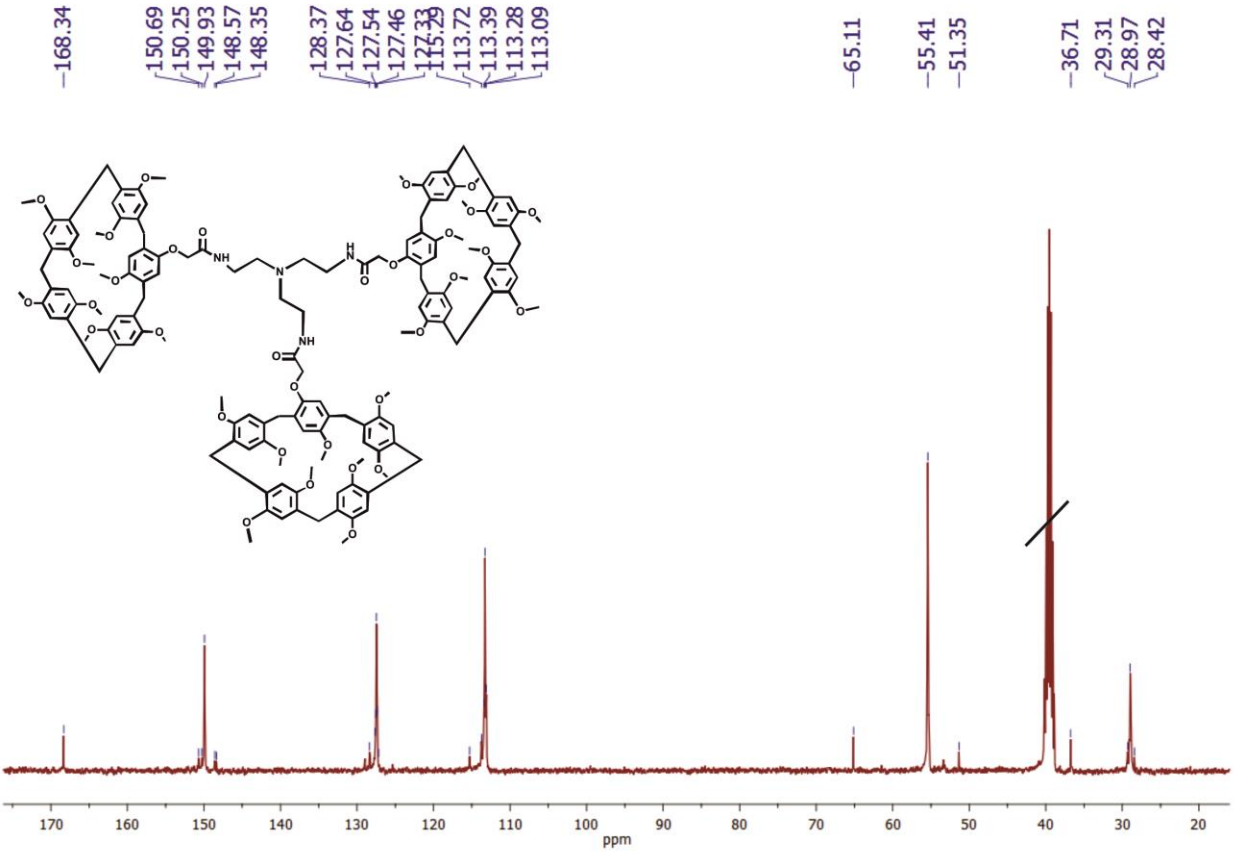

Figure 5S. ^{1}H NMR ($CDCl_3$) spectra of PhTz (red), 4:PhTz complex (blue) and 4 (black).

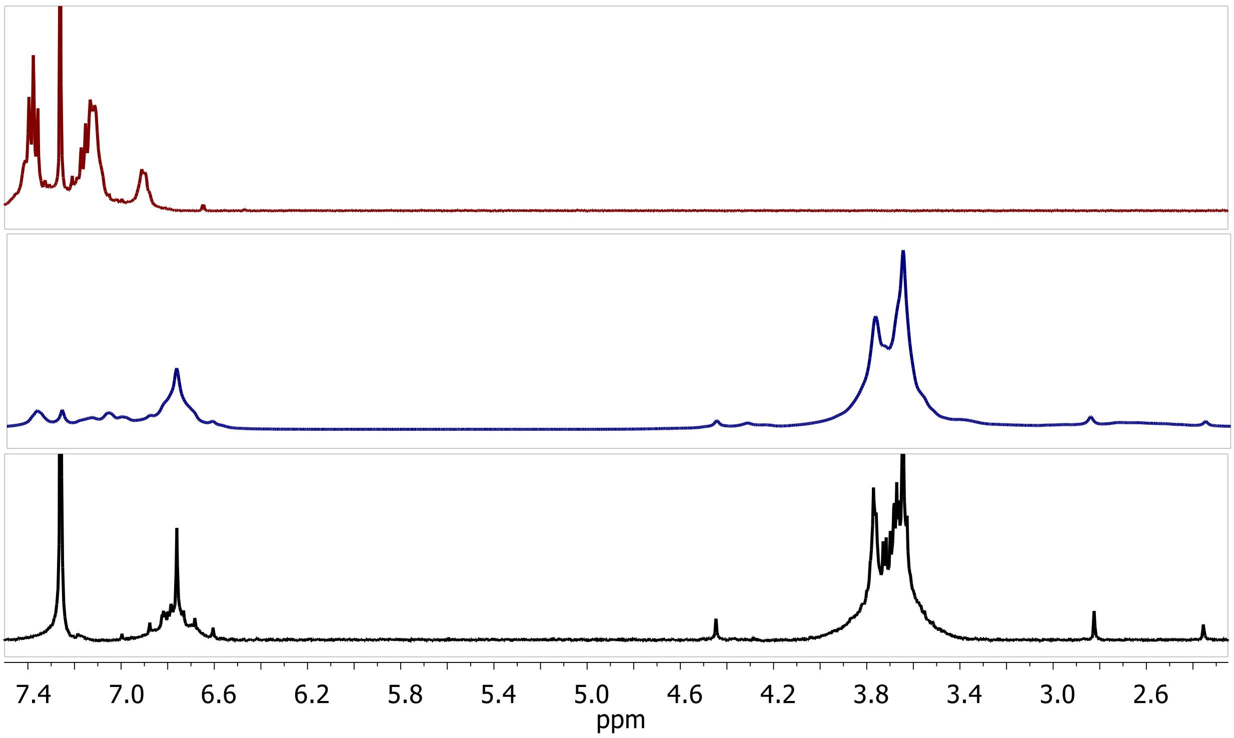

Figure 6S. Fragments of the ^1H NMR ($CDCl_3$) spectra of PhTz (red), 4:PhTz complex (blue) and 4 (black), sections corresponding to signal of aromatic protons (left), ~OCH2CONH~ fragment connecting pillar[5]arene to TREN (center) and N(CH_2CH_2NH) protons (right).

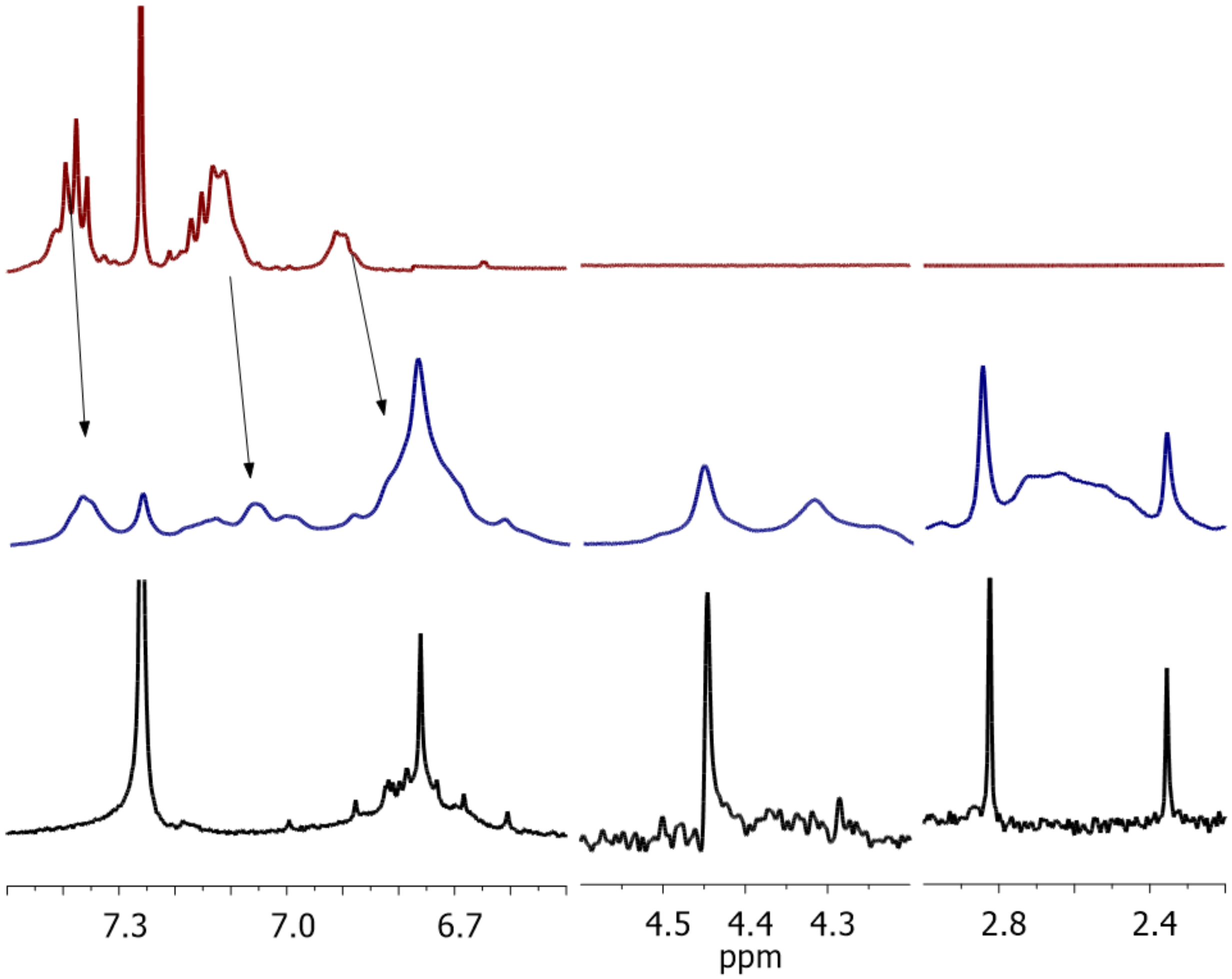

Figure 7S. ^{1}H **NMR (DMSO d$_6$) spectra of PhTz (red), 4:PhTz complex (blue) and 4 (black), section corresponding to signal of amide fragment (left).**

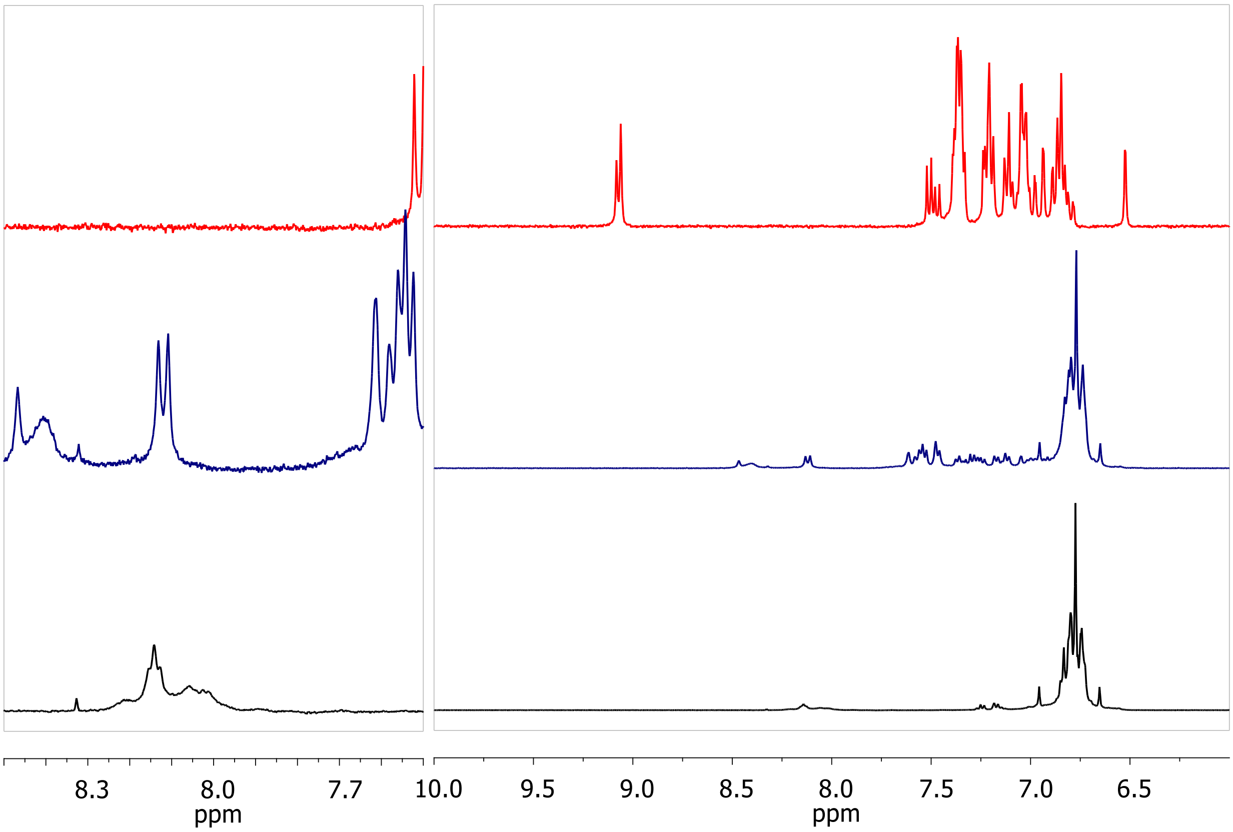

Figure 8S. ^{31}P NMR ($CDCl_3$) spectra of tetrabutylammonium dyhydrogen phosphate ($C=5\times10^{-3}$) (a), mixture of tetrabutylammonium dyhydrogen phosphate and 4 ($C=5\times10^{-3}$) (b) and a mixture of tetrabutylammonium dyhydrogen phosphate, 4, PhTz ($C=5\times10^{-3}$) (c)

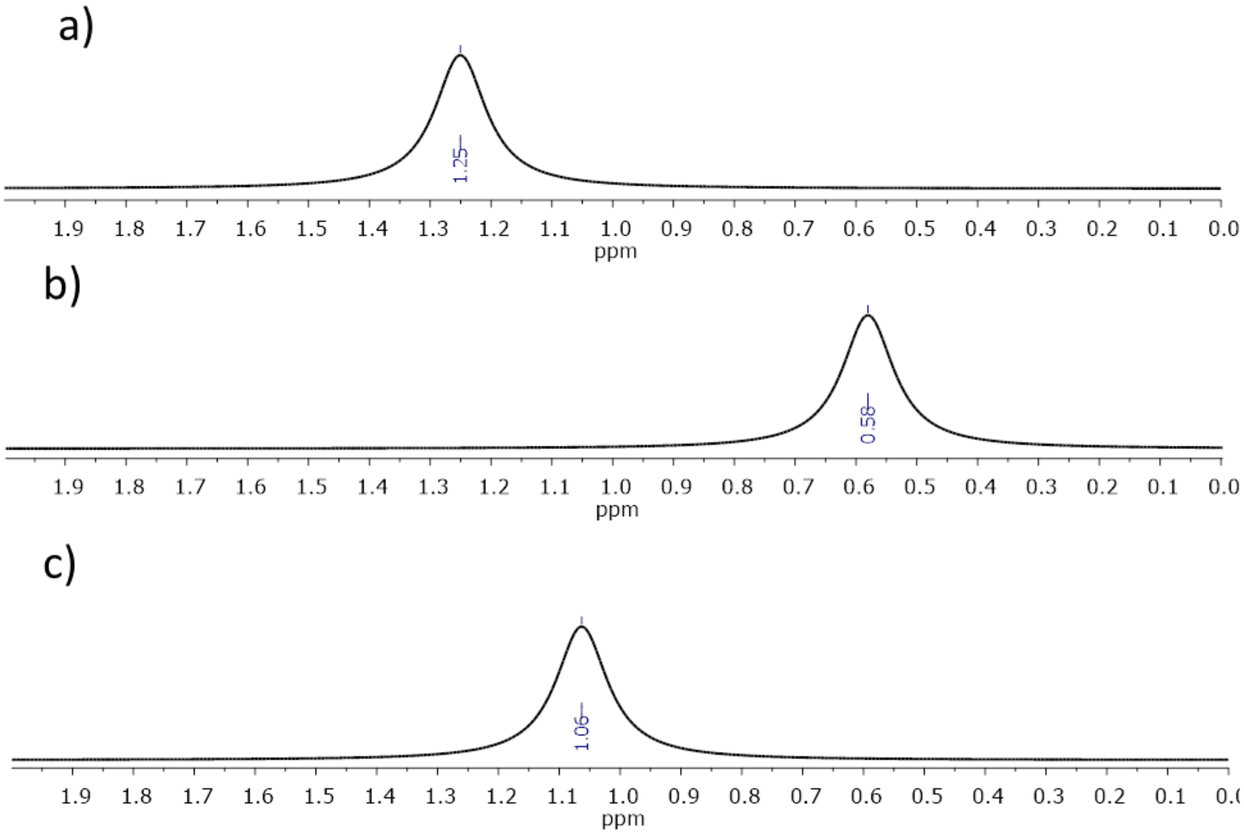

Figure 9S. ^1H NMR (DMSO d_6) spectrum of 3,7-bis(phenylamino)phenothiazin-5-ium iodide, sections corresponding to NH (left) and Ar-H (right) protons

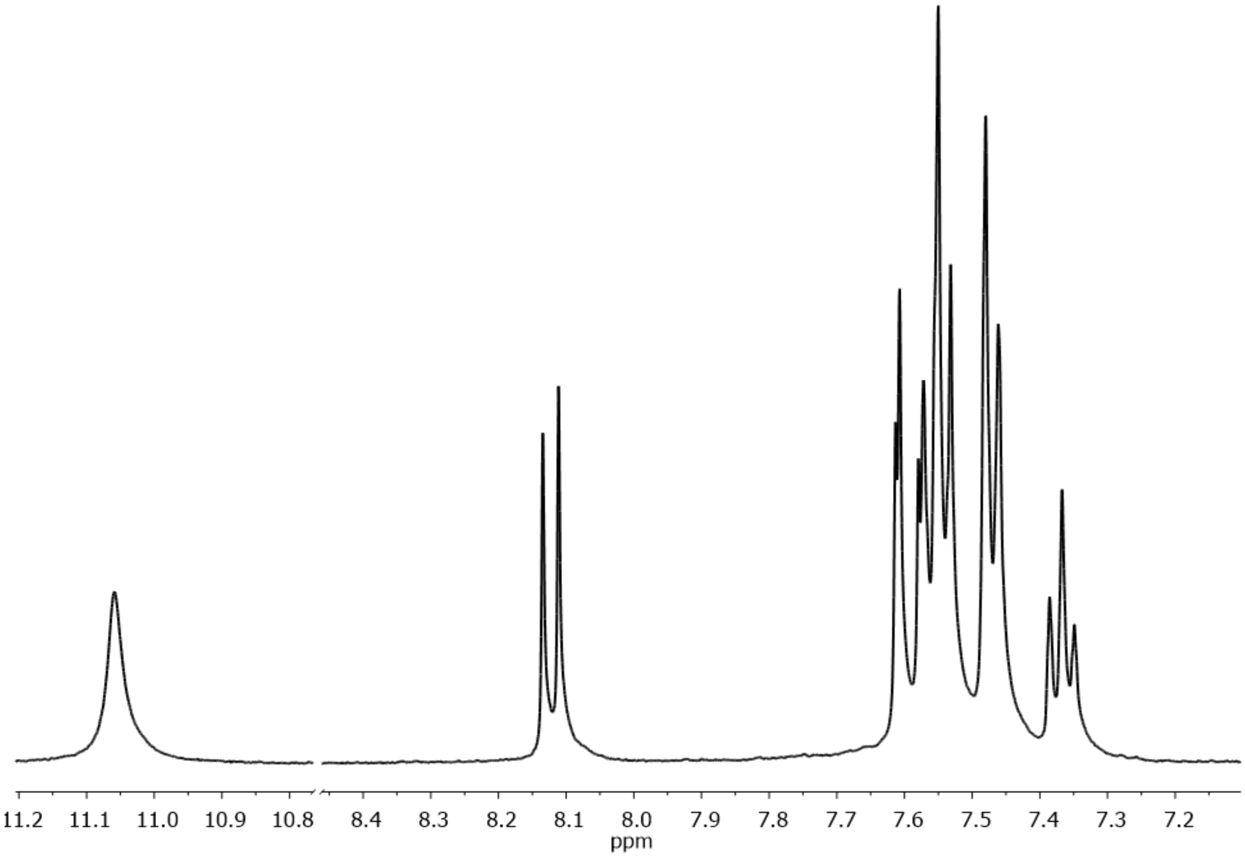

Figure 10S. ^{1}H **NMR (DMSO d_6) spectrum of N-phenyl-3-(phenylimino)-3H-phenothiazin-7-amine (PhTz)**

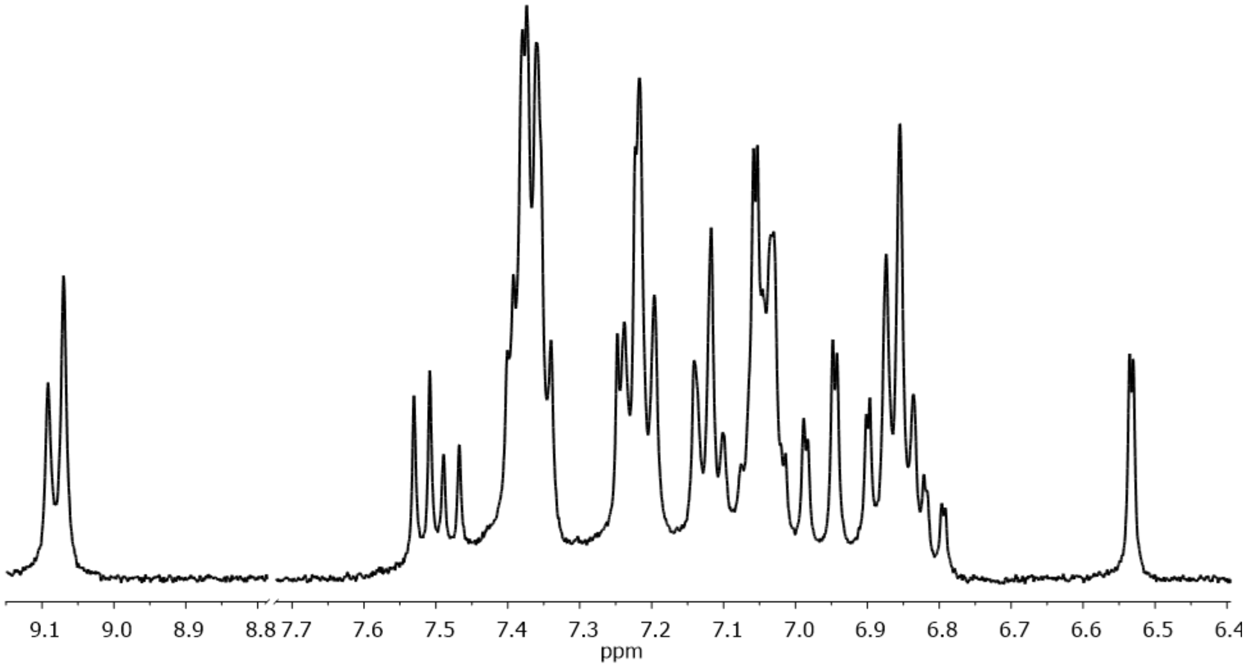

Supplement: Supplementary file 1 [file molecules-24-01807-s001.pdf]
